# Representation of Intimate Partner Violence Against Women in Swedish News Media: A Discourse Analysis

**DOI:** 10.1177/1077801220940403

**Published:** 2020-07-27

**Authors:** Nadja Karlsson, Marisol Lila, Enrique Gracia, Maria Wemrell

**Affiliations:** 1 Lund University, Malmö, Sweden; 2 University of Valencia, Valencia, Spain; 3 Unit for Social Epidemiology, Lund University, Malmö, Sweden; 4 Department of Gender Studies, Lund University, Malmö, Sweden

**Keywords:** intimate partner violence against women (IPVAW), discourse, newspapers, Sweden, Nordic Paradox

## Abstract

Despite being rated as some of the world’s most gender equal countries, Sweden and neighboring Nordic nations show high rates of intimate partner violence against women (IPVAW). As the news media contribute to the shaping of public attitudes, this article pursues a two-step discourse analysis of how IPVAW was represented in seven Swedish newspapers during 2018. Although an individualistic discourse on IPVAW was found to be most prevalent, articles where perpetrators were presented as non-Swedish more frequently contained a structural framing of IPVAW. This confirms previously noted tendencies toward individualization and othering of IPVAW in Sweden.

## Introduction

Globally, one third of all women are estimated to be exposed to physical or sexual intimate partner violence against women (IPVAW) during their lifetime (World Health Organization [WHO], 2019). Contributing to mortality as well as morbidity and injury, this is a serious public health (WHO, 2013) and human rights (European Commission, 2010) issue. As gender inequality is typically assumed to be associated with and function as a predictor of IPVAW rates (e.g., European Institute for Gender Equality [EIGE], 2017; United Nations [UN], 2018), countries with low levels of gender equality are expected to show a high prevalence of IPVAW, and vice versa. However, despite being rated as some of the world’s most gender equal nations (e.g., EIGE, 2017), Nordic countries including Sweden show high IPVAW rates. An EU-wide survey conducted in 2012 found that 28% of women in Sweden reported having experienced physical or sexual violence from a partner (European Union Agency for Fundamental Rights [FRA], 2014), and in 2018, 22 women were killed through intimate partner homicide (IPH; Brottsförebyggande rådet, 2019). This apparent contradiction, the so-called Nordic Paradox (Gracia & Merlo, 2016), of coexisting levels of high gender equality and high IPVAW rates, merits further investigation of IPVAW and of how the phenomenon is understood in Nordic countries.

How the news media represent an issue is important for public understanding and behavior as well as for political responses including available support systems and interventions (Alfredsson et al., 2016; Carlyle et al., 2014; Nayak et al., 2003; van Dijk, 1988). Therefore, this article directs focus toward discursive representation of IPVAW in Swedish newspapers.

### Discourses on IPVAW in Newspapers: An Individual or Structural Issue

A discourse can be described as a way of viewing the world, socially shaped and simultaneously constitutive of the phenomenon in question (Jørgensen & Phillips, 2002), or as language used as a canalization and recreation of social, political, and cultural meaning (Greckhamer & Cilesiz, 2014). The epistemological assumption underlying discourse analysis is thus that the way an event is construed depends on underlying assumptions and world views, as discourses surrounding the same phenomenon, which can vary between persons or groups, enable or constrain different ways of understanding it (Jørgensen & Phillips, 2002). Thus, discourse analysis, and particularly critical discourse analysis (CDA; van Dijk, 2016), accounts for how the use of language constitutes and is informed by sociocultural meaning, with the goal of exposing assumptions and power relations that form different understandings of the world (van Dijk, 2016). CDA often directs attention toward categorizations and their implications, as these can commonly be linked to politically charged formation of Us/Them groups (van Dijk, 1988, 1993a).

With regard to IPVAW, the ecological model developed by Heise (1998) and recommended by the World Health Organization (WHO & Pan American Health Organization, 2012) recognizes an interplay between individual, relational, and sociocultural factors, in causation of IPVAW. Nevertheless, researchers in Sweden (Helmersson, 2017; Holmberg & Bender, 2003; Official Report of the Swedish Government [SOU], 2004:121) and elsewhere (Carlyle et al., 2014) have observed discursive tendencies toward understanding IPVAW as *either* an individual or relational issue *or* a structural problem. When constructed as an individual problem, focus is often on deviancy: on how perpetrators and/or victims or survivors can be considered as deviant from the normative population, in ways that can serve to explain IPVAW (Enander, 2010; SOU, 2004:121). A related discursive strategy termed episodic framing (Carlyle et al., 2014) separates IPVAW events from one another, thus failing to situate them in a larger context. Meanwhile, a more structural discourse on IPVAW places focus on gendered power relations in society and on societal tolerance toward violence against women (Enander, 2010; SOU, 2004:121).

Despite the importance of discursive representation in news reporting for the creation of public consensus about a phenomenon (Meyers, 1996; van Dijk, 1988, 1993a), apart from a study looking at three Swedish newspapers during 2012 (Nationellt Centrum för Kvinnofrid [NCK] & Uppsala University, 2014), limited academic attention has been paid to aggregated representation of IPVAW in Swedish news media. Nevertheless, international research on the representation of IPVAW in news reports has pointed to problematic practices including tendencies toward describing individual aggressors with an emphasis on their positive characteristics, as violent actions have tended toward being excused or justified, or as deviant and different from the majority population (Fairbairn & Dawson, 2013; Leung, 2019; Lloyd & Ramon, 2017; Meyers, 1996; Pepin, 2016; Richards et al., 2014; Venäläinen, 2016). Patterns of victim-blaming, through which the cause of IPVAW is sought in the individual victim’s behavior or characteristics, and of the construction of mutual responsibility for IPVAW, have also been observed (Fairbairn & Dawson, 2013; Hernández, 2018; Leung, 2019; Lloyd & Ramon, 2017; Meyers, 1996; Pepin, 2016; Venäläinen, 2016).

Chesney-Lind and Chagnon (2017) note that although media reports on IPVAW often focus on the details of the events, they tend to omit reference to the larger social context, that is, the extent and causes of IPVAW in society. Individualistic discursive tendencies in media reporting on IPVAW are further expressed in the rare inclusion of IPVAW experts or of reference to resources such as helplines or local shelters (Fairbairn & Dawson, 2013; Hernández, 2018; Leung, 2019; Richards et al., 2014). Acts of IPVAW have often been depicted as cases of “snapping” or as “crimes of passion,” with violence represented as sudden or unavoidable, even when it was premeditated or continuous (Fairbairn & Dawson, 2013; Lloyd & Ramon, 2017; Meyers, 1996; Richards et al., 2014). Such tendencies toward individualization of IPVAW, and of representing the violence as sudden, coexists with research pointing to IPVAW existing across sociodemographic groups (Lundgren et al., 2002; Nybergh et al., 2013) and often taking the form of a continuum of violence (Kelly, 1988).

Along similar lines, the noted report on Swedish media representations of IPVAW (Nationellt Centrum för Kvinnofrid & Uppsala University, 2014) concludes that IPVAW was primarily construed as an individual or relational issue, rather than a structural one. IPVAW was mainly discussed with reference to the perpetrators’ intoxication, confusion, or mental illness, which corresponds with common associations in public discourse between IPVAW and individual factors such as substance abuse, mental illness, or low socioeconomic status (Alfredsson et al., 2016; Brännvall, 2016; SOU, 2004:121) or “particularly vulnerable” groups (Holmberg et al., 2015). This has been related to a recent discursive shift away from a more structural understanding of IPVAW, encompassing attention toward societal systems and relationships, toward a more individualistic one (Holmberg et al., 2015). The report by Nationellt Centrum för Kvinnofrid & Uppsala University (2014) further notes that IPVAW was often described in terms of quarrels or discussions, which in effect placed part of the responsibility on the individual victim. Victim-blaming attitudes have indeed been found to be somewhat prevalent in Sweden, especially among men, as expressed in an understanding of shared responsibility for IPVAW (Alfredsson et al., 2016; Ivert et al., 2017).

In addition, noted tendencies toward othering of IPVAW, of ascribing it to persons or groups labeled as deviant or different from the majority population, have often invoked national, cultural, or racializing categorizations.

### Racialization and IPVAW in News Media

The media have been noted to play a central role in processes of racialization (Alamo-Pastrana & Hoynes, 2018; Bredström, 2003; Drew, 2011; Gans, 2016; Hervik, 2019), through which groups come to be perceived in racial terms (e.g., Hochman, 2019). In the United States, for example, media representations of IPVAW have often excused and justified White perpetrators while pathologizing Black ones (Pepin, 2016). In Europe, although explicit reference to biological race may today be quite rare in public debate, current forms of racism directed against immigrants and minority populations tend to invoke notions of irreconcilable cultural differences and of the superiority of some cultures to others. Biologically based racism has thus largely been replaced by cultural racism (Blaut, 1992), or by cultural differentiation as a means to define ethnic relations (van Dijk, 1993a). Immigrant groups including Muslims have been racialized by being represented as a distinct *Them*, separate from the *Us* of the majority population, on the basis of cultural traits typically represented as threatening (Bonilla-Silva, 2015; El Din, 2019; Gotanda, 2011). Such tendencies have been present in Sweden over the last decades (Bredström, 2003; Deland, 1997), and increasingly so more recently (Hervik, 2019), as seen, for example, in the rise to prominence of the culturally racist (Mulinari & Neergaard, 2015) party Sweden Democrats.

Violence against women, and media representation of it, forms an important location of evocation of cultural difference (Chesney-Lind & Chagnon, 2017; Patil & Purkayastha, 2015; Reimers, 2007), as gender and sexuality have been found to play important roles in the symbolic construction and maintenance of the nation-state and its boundaries and in culturally racist notions of belonging (Bredström, 2003; Mulinari & Neergaard, 2015; Sager & Mulinari, 2018). Thus, through an increasing culturalization of IPVAW within the European Union, notions of ethnicity, race, or culture are articulated alongside notions of gender, constructing an essentially nonviolent and gender equal (White) European *us* contrasted against a violent and gender unequal (non-White) non-European *them* (Montoya & Rolandsen Agustín, 2013). IPVAW is thereby discursively constructed as belonging to the culture or tradition of Others, with so-called honor killings invoking more attention and outrage than IPH committed by members of the majority group (Montoya & Rolandsen Agustín, 2013).

Corresponding tendencies have been identified in Nordic media representations of violence against women (Hervik, 2019; Keskinen, 2012; Reimers, 2007; Saresma, 2019). In Finnish and Danish news reports, Us- and Them-groups have been created through contrasting perceived Muslim identities against a national identity, the latter marked by gender equality, an associated moral superiority, and a relative absence of IPVAW. Similarly, Swedish discourses of gender equality, central to Swedish national identity, have been engaged in the culturalization of IPVAW by construing a Swedish, gender equal and nonviolent *Us* and a non-Swedish, gender unequal and violent *Other* (Brännvall, 2016; Bredström, 2003; de los Reyes, 2003; Gottzén, 2016; Reimers, 2007; SOU, 2004:121). Gender inequality and violence against women in Sweden are thereby represented as largely being due to immigration (Mulinari & Neergaard, 2015). Accordingly, with regard to sexual violence against women, Saresma (2019) notes that “it is only when the rapists come from somewhere else that rape becomes a question of gender equality” (p. 76). Although this discourse is centered on culture, whiteness has been central to perceptions of whether a person belongs to Us or Them (Keskinen, 2012).

## Aim

Against the noted background, the present study aims to investigate how IPVAW was discursively represented in Swedish newspapers during 2018, with regard to the causes of the violence and whether the descriptions vary with the perceived national, ethnic, and/or cultural or religious background of the alleged perpetrators. The study was undertaken in two steps, with the intention to answer two related but separate research questions:

**Research Question 1:** What main type of discourse on IPVAW is used in news articles?
**Research Question 1a:** Do the articles use individualistic or more structural discourses on IPVAW?
**Research Question 1b:** Does the discursive representation vary with the presented or perceived national, ethnic, religious, and/or cultural background of the alleged IPVAW perpetrators?
**Research Question 2:** What explanatory models for IPVAW are presented in the articles?

IPVAW is here understood, in accordance with the UN (2006), as sexual, psychological, physically, or economically coercive acts against women by a current or former partner, including online violence and violence facilitated by information and communications technology (Office of the UN High Commissioner for Human Rights, 2018), without her consent. The term “victim,” not “survivor,” is used, as the data include lethal violence.

## Method

### Data Collection

Articles were gathered from seven Swedish newspapers, namely two national daily newspapers: *Dagens Nyheter* (*DN*) and *Svenska Dagbladet* (*SvD*); two national evening newspapers: *Aftonbladet* and *Expressen*; and three regional newspapers: *Göteborgsposten* (*GP*), *Sydsvenskan* (*SvD*), and *Västerbottens-Kuriren* (*VK*), during 2018. The newspapers were chosen with consideration of type, geographic range, and political affiliation, to include national and more local or regional papers; morning and evening papers; papers from the northern, southern, and central parts of Sweden; and papers with social democratic, conservative, and liberal affiliation, the latter being most common due to an overrepresentation of liberal newspapers in Sweden.

The data were collected via the search engine Retriever Research, using the search terms (violence [våld*] OR abuse [*misshand*] OR violation of integrity [*fridskränkning*] OR murder [mord*] OR threat [hot*] OR honor [heder*]) NEAR (relationship [relation*] OR partner [partner*] OR couple relationship [parrelation*] OR relationship [förhållande*] OR cohabiting [sambo*] OR family [familj*] OR boyfriend [pojkvän*] OR husband [make*] OR girlfriend [flickvän*] OR wife [fru*] OR wife [hustru*] OR woman [kvinn*]). These terms were selected to enable the inclusion of a wide range of articles, which were limited to printed material, excluding online publications to avoid doublets and paywalls.

The identified articles were assessed and included if they met the inclusion criteria regarding (a) *date*: The articles were published during 2018; (b) *type of article*: Only articles based on or describing IPVAW cases were included (articles broadly discussing IPVAW without referring to specific cases or events were thus excluded, as were reports making brief references to cases but not focusing on specific ones); (c) *gender*: This study includes cases in which the alleged victim was a (cisgender or transgender) woman, although the suspected aggressor could be of any gender (while IPVAW usually refers to violence against adult women, girls were included); (d) *relationship*: Articles explicitly defining the relationship between the abuser and the abused, describing the event as taking place in a home environment, or using judicial terms denoting IPVAW (e.g., *kvinnofridskränkning*) were included; and (e) *location*: The cases needed to have a connection to Sweden.

If an article was published in more than one newspaper, all versions were included to provide an accurate account of the frequency of articles depicting IPVAW in certain ways.

### Study Design and Discourse Analysis

We applied a two-step discourse analysis, inspired by CDA (van Dijk, 1993b). While CDA studies can be either qualitative or quantitative, performed deductively or abductively (van Dijk, 2016), this study used a combined approach. A quantitative, primarily deductive analysis was thus followed by a selective, abductive, and qualitative study.

#### Step 1: Quantitative analysis

The quantitative analysis addressed the first research question, regarding the prevalence of individualistic and structural discourses in news reports on IPVAW. The coding of the material, drawing on Leung (2019), Pepin (2016), and Fairbairn and Dawson (2013), included (a) *the type of violence* in focus (e.g., abuse or murder), (b) *gender of the aggressor*, (c) the potential inclusion of *national/ethnic/religious/cultural background*, and (d) any *recognition of IPVAW as a societal problem* (e.g., statistics or expert statement). The unit of analysis was the article and not the case (Fairbairn & Dawson, 2013). The categories of the variables are defined in Table 1. However, the categorization of (c) potential inclusion of national/ethnic/religious/cultural background merits some further description.

**Table 1.** Categorization of Quantitative Variables Related to Media Representation of IPVAW in Sweden, 2018.

Variables	Definition	Merged categories
Categories		
Type of crime in focus^a^		
Lethal violence	Murder, causing another’s death, and crimes against the peace of the tomb where the dead body had been handled in such a way that cause of death was impossible to determine	
Physical violence	All forms of physical violence, including but not limited to assault and attempted murder	
Sexual violence	All forms of sexual violence, including but not limited to rape and forced or child-marriage	
Psychological violence and controlling behavior	All forms of psychological violence and controlling behavior, including but not limited to threats and stalking	
Material violence	Violence aimed at, or causing harm to, personal property	
Undefined violence	Violence asserted but not defined, for example, “violence of (a woman’s) integrity” (kvinnofridskränkning) of varying degrees, where the offenses making up the crime classification are not presented	
Gender of the aggressor^b^		
Male	Including male pronouns, titles (e.g., husband), and names	
Female	Including female pronouns, titles (e.g., wife), and names	
Not defined	No gender-identifying information presented	
Potential inclusion of national/ethnic/religious/cultural background		
Swedish-presenting	Defined as Swedish, Swedish-sounding name, and/or White-presenting with no other identifier	
Non-Swedish, White/European-presenting	Pronounced European or White-presenting with non-Swedish-sounding name	
Other-presenting	Defined connection to “other” area than above; presented as non-Swedish with no further definition; religion other than Christianity; non-Western-sounding name; and/or non-White-presenting	
Type of discourse		
Structural	The article includes or is placed in connection to statistics, general information, or expert comments on IPVAW or related subjects^c^	Structural
Semi-structural	The article mentions relevant terms or placed the specific case in relation to other cases of violence against women, not necessarily IPVAW, or ideas about IPVAW^d^	
Individual	The article does not include any of the above components	

*Note.* IPVAW = intimate partner violence against women.

a Economic violence did not receive its own category but was categorized under the other ones. ^b^No other genders were reported. ^c^This is a rather low-set criteria for a structural discourse as no actual societal or power analysis is required. ^d^Ideas that women lie were not included here, as these refer to the phenomenon of alleged false accusations rather than to the phenomenon of violence against women.

When comparing how White and Black perpetrators of IPVAW are described in the United States, Pepin (2016) focuses on celebrities, thus enabling the use of external sources to identify the perceived “race” of the individuals. In this study, this was rarely possible. Only when an article *de facto* describes a person as, for example, “Swedish” or “immigrant from XX,” may any actual conclusions be drawn regarding their background. However, persons are rarely described as “Swedish” in the articles. Therefore, when no such information was provided, images and names were used for categorization. Although appearances and names are poor indicators of national/ethnic/religious/cultural background, previous studies show, as noted, that despite culturalizing framings of Us and Them, skin color is still an important indicator as to which group someone will be perceived as belonging (Keskinen, 2012).

Our coding of national/ethnic/religious/cultural background was conducted in steps. The first concerned indications of country/nationality and included Sweden, Western Europe/North America/Australia, Eastern Europe, Europe undefined, Other areas, and non-Swedish undefined. In the second step, indications of religion were noted. The third step included names, coded as Swedish-sounding and non-Swedish-sounding, and images coded as White-presenting, non-White-presenting, and inconclusive. Coding of names and images was done by two coauthors separately. Names overruled images, and when an image was inconclusively coded, it was not used as a basis for categorization. The final categorization was made on the basis of the three steps combined and distinguished between three categories: (a) Swedish-presenting; (b) non-Swedish-, White/European-presenting; and (c) “Other”-presenting. This reflects previous categorizations of Us and Them groupings in Nordic countries (Keskinen, 2012; Montoya & Rolandsen Agustín, 2013; van Dijk, 1993a).

Discourse types were categorized as structural, semi-structural, or individualistic (see Table 1). An article was considered to use a structural discourse when it encompassed any form of contextualization of the IPVAW case, through inclusion of, for example, statistics, expert commentary, contact information to support organizations such as women’s shelters and/or general information about IPVAW or related subjects (i.e., other types of violence against women or girls, including sexual harassment, so-called honor-based violence [HBV], wife import, child-marriage, and/or forced marriage), as inclusion of this type of content provides recognition of the societal and structural context of IPVAW (Fairbairn & Dawson, 2013). It should be noted that the definition of structural discourse used in this study does not require societal or power analysis or reference to factors such as gender norms or inequalities (compare, for example, Holmberg et al., 2015). An article was coded as using a semi-structural discourse when it included relevant or related terms such as IPVAW, men’s violence against women, “honor,” or comparisons with other or generalized cases of IPVAW or other violence against women. An article lacking any of the above was coded as using an individualistic discourse.

The search results were reviewed 3 times, ensuring that the same inclusion criteria were applied to all articles. NVivo 12 Plus software was used for coding, and the results were entered into IBM SPSS Statistics 25 where chi-square tests were run.

#### Step 2: Qualitative analysis

In the qualitative part of the analysis, which addresses the second research question regarding attributed causes of or explanatory models for the depicted IPVAW, articles from January through April 2018 were coded abductively. This coding was primarily kept on a manifest level and structured under subcategories, categories, and themes, inspired by the abstraction process outlined by Elo and Kyngäs (2008; see Table 2). The entire articles were not coded but only the parts that were relevant to the research question. Discursive explanatory models were identified through a focus on attributed responsibility for the IPVAW and on “overcompleteness,” that is, inclusion of details not necessary for recounting the event, which could thus be considered explanatory (Meyers, 1996; van Dijk, 1993a).

**Table 2.** Examples of Coding Conducted When Exploring Discourses on Individual Explanatory Models Related to Media Representation of IPVAW in Sweden, 2018.

Meaning unit^a^	Code	Subcategory	Category	Theme
“[Perpetrator] has stated in interrogations that on the day of the murder he fell out with [victim] . . . The quarrel escalated and ended up with him [violent action]”	Fight having escalated	Couple having conflict(s)	Relational causes	Personal life situation
“. . . the man has insisted that he [violent action] after getting drunk . . .”	Having been drunk^b^	Substances in direct relation to event	Indicating use of drugs and/or alcohol	Personal life situation
“It is not uncommon that women wrongly claim to have been assaulted and threatened . . .”	Women lie about abuse		Insinuating or claiming false accuses	Proposing alternative theories

*Note.* IPVAW = intimate partner violence against women.

Subcategories were developed from several similar codes, categories consist of several related but different subcategories, and the themes represent which types of explanatory model are present in the articles. Due to the character of themes in this study, it is possible for a theme to comprise categories lacking subcategories.

a Reduced, masked, and translated to sustain anonymity. ^b^ When no person is indicated, the codes relate to the perpetrator. When a code concerned the victim, it was indicated in the code.

All articles from January to April were included, and all categories and codings were double-checked. Again, NVivo 12 Plus software was used for the manual creation of codes, subcategories, categories, and themes.

## Ethical Considerations

The data were collected from material already published, but for the purpose of anonymity, no names or other identifiers are mentioned in this article. Quotations are used only to showcase the coding process, through inclusion of the authors’ (masked) translations and not the original text. Furthermore, as due diligence is necessary in research that risks stereotyping or marginalizing individuals or groups, it should be noted that this study in no way intends to construct differences between groups. Rather, the aim is to investigate whether a difference *is made* between groupings of people in Swedish media discourse and in that case to problematize it. The project was approved by the Swedish Ethical Review Authority (Dnr 2019-00221).

## Results

The search and selection process generated 4,391 articles, 325 of which fulfilled the inclusion criteria and were thus analyzed. An additional two articles, excluded from the quantitative analysis due to a glitch in the search engine Retriever Research rendering the context of the texts invisible, were used in the qualitative part of the study. The sources included paragraphs, shorter and longer articles, reportages, and debate articles.

The noted perpetrator was a man in 319 articles, a woman in two articles, and not identified by gender in four. Cases where the victim had died were described in 147 articles (45%), making this the most commonly reported form of IPVAW. Most articles (*n* = 215) did not include or included only inconclusive information on nationality, ethnicity, or religion.

In 48 articles, a country/nationality was indicated for the perpetrator, 16 of which described them as Swedish. Eastern Europe and an undefined European country were indicated in two articles each, non-Swedish undefined heritage in eight, and other countries/areas in 20 articles. Including names, images, and stated/indicated religion, it is likely that, out of those which provide some kind of ethnic/national/religious identification, 63 articles included someone who would likely be perceived as Swedish by the newspaper audience; 11 referred to someone non-Swedish-, White/European-presenting; and 37 included someone from or with connections to another area. Names and images primarily played a role in identifying people who would likely be perceived as Swedish, whereas stated or indicated religion was more often an indicator of someone likely to be perceived as Other. A few more articles included images, though these were inconclusively coded, and it is thus likely that the perception of these individuals would vary between readers.

In the majority of the articles, there was no recognition of structural aspects of IPVAW, and individual cases were not positioned in a larger societal context. Twenty-one of the articles provided information designating them to a more structural discourse, whereas 56 articles used wordings or comparisons meriting categorization as semi-structural. A total of 248 articles, or 76% of the sample, were categorized as using an individualistic discourse.

### Structural and Semi-Structural Discourses

As seen in Table 3, of the 21 articles using a more structural discourse, 12 included identifying information, half of which depicted the perpetrator as Swedish-presenting and the other half as Other-presenting. This means that articles with an Other-presenting perpetrator make up 28.6% of all the articles using structural discourse, while only comprising 11.3% of the total number of articles. It also means that 16.2% of the articles with an Othering description of the perpetrator use a structural discourse, whereas articles with a structural discourse make up only 6.4% of the total number of articles. There is thus an overrepresentation of Other-presenting perpetrators in articles using a structural discourse. A chi-square test indicated a significant association between represented identity and article discourse (*p*-value = .018), with a medium effect size (Cramer’s *V* = 0.231). However, as several cells have an expected count less than five, the chi-square test should be interpreted with caution.

**Table 3.** Crosstabulation of Represented Identity and Article Discourse (Structural, Semi-Structural, or Individual) in Articles Depicting Intimate Partner Violence Against Women in Seven Swedish Newspapers, 2018.

			Article discourse			
	Represented identity		Individual	Semi-structural	Structural	Total
	Swedish-presenting	Count *n* (%)	50 (78)	8 (13)	6 (9)	64
		% in article discourse	67	33	50	57,70
	Non-Swedish-, White/European-presenting	Count *n* (%)	8 (73)	3 (27)	0 (0)	11
		% in article discourse	11	13	0	9,90
	“Other”-presenting	Count *n* (%)	17 (47)	13 (36)	6 (17)	36
		% in article discourse	23	54	50	32
Total		Count *n* (%)	75 (68)	24 (22)	12 (11)	111

All but one of the articles with an Other-presenting perpetrator using a structural discourse mentioned the so-called HBV, whereas the remaining one provided statistics on child-marriage. Some of the articles contained additional information on IPVAW and men’s violence against women.

Furthermore, and as seen in Table 3, 36.1% of the articles that Othered the perpetrator used a semi-structural discourse, whereas for non-Swedish-, White/European-presenting perpetrators, the share was 27.3%, and for Swedish-presenting, 12.5%. A total of 23.2% of the semi-structural articles also construed the perpetrator as Other-presenting, which corresponds to 54.2% of the semi-structural articles with any identifying information. Once again, articles with Other-presenting perpetrators were overrepresented.

When the structural and semi-structural categories were conflated into one (see Table 4), of all articles presenting the perpetrator as Other, 50% used a semi-structural/structural discourse. This can be compared with 21.9% and 27.3% of articles referring to Swedish-presenting and non-Swedish-, White/European-presenting, respectively. A chi-square test indicated a significant association between represented identity and article discourse (*p* value = .014), with a medium effect size (Cramer’s *V* = 0.277).

**Table 4.** Crosstabulation of Represented Identity and Article Discourse (Structural or Individual) in Articles Depicting Intimate Partner Violence Against Women in Seven Swedish Newspapers, 2018.

			Article discourse		
	Represented identity		Individual	Semi-/Structural	Total
	Swedish-presenting	Count *n* (%)	50 (78)	14 (22)	64
		% in article discourse	66	40	58
	Non-Swedish-, White/European-presenting	Count *n* (%)	8 (73)	3 (27)	11
		% in article discourse	11	9	10
	“Other”-presenting	Count *n* (%)	17 (47)	19 (53)	36
		% in article discourse	24	51	32
Total		Count *n* (%)	76 (69)	35 (32)	111

### Explanatory Models

Abductive coding of articles from January through April (108 articles), encompassing all articles regardless of whether they were previously coded as structural, semi-structural, or individual, provided information about common explanatory models used to explain IPVAW. Table 2 provides examples of how the analysis translated meaning units into themes.

Four themes emerged (see Table 5). One was *no explanation*, as no additional information and thus no explanatory models were included in the article. A second theme named *proposing alternative theories* was comprised by the alleged perpetrators’ explanations and claims (including the subcategories of *blaming someone else*, *claiming memory loss or unconsciousness*, *insinuating/claiming false accusations*, and *stating that the act was noncriminal or without intent*), alongside quotes from lawyers or lay judges claiming or insinuating that women lie about violence (*insinuating/claiming false accusations*). Thus, this theme did not try to explain the IPVAW but rather to disprove it.

**Table 5.** Model of Subcategories, Categories, and Themes of Individual Explanatory Models Developed From a Qualitative, Abductive Coding of Articles Depicting Intimate Partner Violence Against Women in Seven Swedish Newspapers, 2018.

Subcategories	Categories	Themes
Family of perpetrator having a role	Families playing a role	Collective responsibility
Woman’s family holding part of the responsibility		
Social institutions failing with regard to the perpetrator	Societal failure	
Social institutions failing the victim		
Criminal history and connections	Criminal record and violence against women and others	Personal life situation
History of violence and being considered dangerous		
Violence caused by previous violence		
Difficult childhood	Having had a difficult childhood/life	
General difficulties in life		
Habits and addictions	Indicating alcohol/drugs	
Substances in direct relation to event		
(Mental) poor health and/or disorder	Insinuating (mental) health or social issues	
Malfunctioning social life		
Not having mental health issues		
Couple having conflict(s)	Relational causes	
Gender-related conflicts		
Relationship as cause		
Unequal power relation		
Victim’s actions		
Deviating from (Swedish) norms	Othering	
National background other than Swedish		
Religious or cultural background other than Swedish norm		
Violence not seeming in concordance with personality/temper	Personality and temper	
Violence seeming in concordance with personality/temper		
Economic status or concerns	Socioeconomic status	
Professional title or work status		
Social status		
	Blaming someone else	Proposing alternative theories
	Claiming memory loss or unconsciousness	
	Insinuating/claiming false accusations	
	Stating act as noncriminal or without intent	
		No explanation

A third theme was *collective responsibility.* This included the category of *societal failure*, where institutions such as the police, health care, or social services were noted to have known about the ongoing violence but had failed to act, underestimated the threat from the perpetrator, or failed the perpetrator previously in life, for example, by not having intervened in family violence in their childhood (*social institutions failing the victim* and *social institutions failing with regard to the perpetrator*). This theme also encompassed *families playing a role*, which included, for example, the perpetrator’s family believing in (their son’s) denial of violence (*family of perpetrator playing a role*) and a woman or girl’s family being part of, and enabling, the violence or attempting to help her (*woman’s family holding part of the responsibility*).

By far the most common theme, however, is *personal life situation*, which locates explanations for IPVAW in the life situation or circumstances of the individual. The categories included in this theme are noted below.

#### Personal life situation as explanation

Previous or current *use of alcohol or drugs* was a recurring subject, most often with reference to the perpetrator but also at times to the victim, including both *habits and addictions* and use of *substances in direct relation to the event*.

Articles further pointed to the perpetrator’s *(mental) health and/or social issues*. The most common subcategory was *poor (mental) health and/or disorders*, ranging from references to mental or personality disorders to symptoms and insinuations of such issues and to general health concerns. The importance of mental health as an explanatory model is underscored by the inclusion, in several articles, of the subcategory of *not having mental health issues*, which reveals a potential expectation of such. A few articles included *malfunctioning social life*.

Another category is that of s*ocioeconomic status*, ranging from describing *professional title/work status* to portraying *economic status or concerns* or power position in society (*social status*). In the category of *Othering*, details about perpetrators describing them as non-Swedish were noted (*national background other than Swedish, religious or cultural background other than Swedish norm*) or, though quite uncommon, as *deviating from (Swedish) norms*, such as relaying the perpetrator having a high number of children or wives.

Under the category of *having had a difficult childhood/life*, some articles attended to the childhood and previous life of IPVAW perpetrators, at times including images of them as children (*difficult childhood; general difficulties in life*). Such noted difficulties included experiences of domestic violence, parental drug use, or bullying.

Other categories include *personality and temper*, where the perpetrator’s personal traits, for example, anger or coldness, were an indicated reason for the IPVAW (*violence seeming in concordance with personality/temper*), though the subcategory of *violence not seeming in concordance with personality* simultaneously offered examples of persons expressing surprise at the IPVAW due to descriptions of the man as, for example, calm.

Recounting *the perpetrator’s criminal record and previous violence against the same woman, other women, or other people* was a rather common approach, making other crimes a part of the explanatory model. A lack of previous criminal record was sometimes reported (*criminal history and connections; history of violence and being considered dangerous*). In some cases, the IPVAW appears to have been caused by previous violence, for example, with the aim of making the victim retract a police report (*violence caused by previous violence*).

The category *relational causes* revolved around characteristics of the violent relationship. The majority of the subcategories involved reciprocity and the victim’s role in the IPVAW, encompassing descriptions of the relationship as conflicted, mutually destructive, or tempestuous, or indications of a quarrel prior to or as a part of the IPVAW (*couple having conflict[s]*), as well as questioning of the victim’s behavior or actions (*victim’s actions*). The latter included staying in contact with the perpetrator after ending the relationship and not wanting to take part in police investigations. At times, the relationship itself, or a feature thereof, such as the perpetrator having a new girlfriend, was mentioned (*relationship as cause*). Deviating from the other subcategories are those of *gendered conflicts* (a very small subcategory) and *unequal power relations*, where attention was paid to a more one-sided conflict. Here the victim is noted to be in a position of dependency, or the perpetrator is said to have had more control in the relationship. This ranged from the man being described as physically bigger and stronger to a recognition of the situation of a foreign woman dependent on her relationship with the perpetrator for her residency permit. In some articles, this was discussed as a structural problem and in others as a feature of the particular relationship. Thus, although some of these articles were coded as individualistic in the quantitative analysis, some were designated as semi-structural/structural.

## Discussion

IPVAW is commonly assumed to be associated with gender inequality (e.g., EIGE, 2017; UN, 2018). However, despite being rated as some of the most gender equal in the world, Sweden and neighboring Nordic countries show high survey-reported IPVAW rates. While not providing an explanation for this Nordic Paradox (Gracia & Merlo, 2016), this study shows that the assumed link between gender inequality and IPVAW is not commonly emphasized in Swedish news articles, as an individualizing discourse on IPVAW was found here to be much more predominant than a more structural understanding of IPVAW, such as that associated with feminist and women’s shelter movements (e.g., SOU, 2004:121). These results align with the observation that although gender inequality has been linked to IPVAW in Swedish public discussion since the 1970s, it coexists with and is often deprioritized in the face of more individually or relationally oriented explanatory models (e.g., Wemrell et al., 2019). It has further been noted that assumptions of gender equality can obscure the presence of IPVAW and gender inequalities in Sweden, while validating individualizing discourses and also making it difficult for women and men to recognize themselves as IPVAW victims and perpetrators and to seek and receive support (Wemrell et al., 2019). Meanwhile, this study shows stronger tendencies toward using a more structural discourse when news articles represented IPVAW perpetrators as non-Swedish. This confirms the presence of tendencies in Swedish public discourse toward othering of IPVAW (e.g., Gottzén, 2016) through associating it with structural patterns located in non-Swedish rather than Swedish contexts.

It should be reemphasized that the criteria used in this study for categorization as structural discourse were set relatively low, not requiring any societal or power analysis (compare, for example, Holmberg et al., 2015). Nevertheless, and in correspondence with the findings of a previously noted study (Nationellt Centrum för Kvinnofrid & Uppsala University, 2014), the majority of news articles did not position the reported case in any societal context. It would not be unreasonable to include IPVAW expert commentary, statistics, or contact information for shelters or helplines, at least in lengthier articles, and although such information was occasionally present, it was not standard practice. It has been suggested that apart from supporting a structural understanding of IPVAW, such inclusion could counter isolation and potentially increase help-seeking behavior among victims and perpetrators (Carlyle et al., 2008; Nutbeam et al., 2010), while also increasing the awareness of IPVAW among the general public, potentially making people more prone to react to others’ behavior or victimization, and affect available public interventions (Alfredsson et al., 2016; Carlyle et al., 2014; Fairbairn & Dawson, 2013).

The preponderance of explanatory models focused on various aspects of the *personal life situation* of the perpetrator, such as the use of alcohol or drugs, mental illness, socioeconomic status, criminal history, or personality traits of the perpetrator which is in line with previous observations from Sweden (Nationellt Centrum för Kvinnofrid & Uppsala University, 2014) and elsewhere (e.g., Pepin, 2016). Reference to some of these phenomena, notably the mental health status (Pepin, 2016) or difficult childhood (Isaacs & Mthembu, 2018) of perpetrators, has been interpreted as serving to create sympathy for and excuse violent behavior. Opinions on whether to report on use of alcohol have differed, as some researchers consider underreporting of alcohol consumption problematic (e.g., Carlyle et al., 2008), whereas others argue that it may serve to excuse the violence (e.g., Isaacs & Mthembu, 2018; Nationellt Centrum för Kvinnofrid & Uppsala University, 2014; Pepin, 2016; Venäläinen, 2016) and that drugs and alcohol may be associated with but not cause IPVAW (Fleury et al., 2000). References to the personality and temper of abusers have also been noted to pathologize and excuse IPVAW (Isaacs & Mthembu, 2018; Nationellt Centrum för Kvinnofrid & Uppsala University, 2014). Such tendencies toward pathologizing or excusing IPVAW allow readers to maintain a distance from IPVAW and understand it as an individual rather than a societal problem (Meyers, 1996), thus not requiring societal change.

Contrasting with previous research observing that IPVAW is often described as one-time events of “snapping” (Fairbairn & Dawson, 2013; Meyers, 1996), included articles commonly referred to or even emphasized the perpetrators’ history of violence. However, descriptions of events as quarrels or fights, or implications that a quarrel caused the IPVAW, were also common. This communicates a vagueness of attributed responsibility, moving a part of the agency from the perpetrator onto the victim, that is, victim-blaming (Meyers, 1996; van Dijk, 1993a), which has also been identified in previous studies (e.g., Nationellt Centrum för Kvinnofrid & Uppsala University, 2014; Pepin, 2016). A similar effect is produced by the questioning of a victim’s staying or meeting with her (ex-)partner. The assumption that an IPVAW victim is freely and easily able to leave a relationship (Lloyd & Ramon, 2017) and that divorce will put an end to IPVAW (Fleury et al., 2000) can be contrasted with the noted heightening of risk for (lethal) IPVAW during the process of separation (e.g., Ekbrand, 2006).

Unequal distribution of power in a relationship, which is a recognized risk factor for IPVAW (Heise, 1998), was sometimes noted. Representations of such inequality ranged from descriptions of the man as being physically bigger to a recognition of the state-created dependency of immigrant women on their partners for residency permits. Still, such power imbalances were often mentioned only with regard to the individual case and not to power inequalities in society.

With reference to explanatory models grouped under the category of *collective responsibility*, attention to criminal record has in some studies been understood to blame the justice system, rather than the perpetrator, for not preventing IPVAW (Isaacs & Mthembu, 2018). Indeed, in this study, the *societal failure* category shifts part of the responsibility for IPVAW onto institutions such as the police or health care. Furthermore, representations of the family of the victim sometimes trying to help and sometimes forming part of the problem are in line with a growing area of research into the positive and negative impacts on the victims’ and/or perpetrators’ families, on IPVAW, and the victim’s possibility to leave the relationship (Hydén, 2015; Sandberg, 2016).

Explanatory models grouped under *proposing alternative theories*, including alleged perpetrators’ diversion of blame, correspond with research pointing to tendencies of denying responsibility for violence among Swedish IPVAW perpetrators. Such evasion of responsibility has typically been affected through reference to the perpetrator’s personal background, to particular circumstances surrounding the violence (Boethius, 2015; Gottzén & Jonsson, 2012), or to the culpability of the victim (Boethius, 2015; Gottzén & Jonsson, 2012; Håland et al., 2016). In general, a resistance toward identifying with IPVAW perpetration has been noted among Swedish offenders (Boethius, 2015; Edin & Nilsson, 2014; Gottzén, 2016; Håland et al., 2016). Thus, despite gendered perspectives on IPVAW in Swedish public discussion, and in alignment with individualizing and victim-blaming discourses used in news media, individually oriented explanations and tendencies toward victim-blaming are common among IPVAW perpetrators in Sweden. Furthermore, references to the perpetrators’ claims of memory loss resonate with research showing a pattern of perpetrators attributing IPVAW to alcohol, blackouts, or loss of control (Boethius, 2015; Gottzén & Jonsson, 2012; Hydén, 1992). Accordingly, the process of facing up to one’s own acts of IPVAW has been described by Håland et al. (2016) as moving from denial to recognition of the self as a perpetrator, in a process that “can be understood as an encounter with the Other as one’s own mirror image” (Gottzén & Jonsson, 2012, p. 156).

### The IPVAW Perpetrator as Other

Although the news articles were categorized as structural when they included some form of contextualization of the IPVAW case, it is noteworthy that such contextualization was not evenly distributed among articles portraying perpetrators as Swedish-presenting or non-Swedish-presenting. This study shows a stronger tendency toward including statistics, expert comments, key terms, or comparisons in articles presenting the perpetrator as non-Swedish. Thus, whereas many news articles leaned toward individualizing, pathologizing, and even excusing violence, they were more likely to present IPVAW as a structural issue in cases where the perpetrator was understood as non-Swedish. Tendencies toward representing IPVAW as “other” (e.g., de los Reyes, 2003; Keskinen, 2012)—as a deviancy when committed by one of Us but more of a collective or cultural issue when committed by one of Them—were present. This is in line with discourses of culturalization, through which IPVAW is assumed to belong to certain cultures (Montoya & Rolandsen Agustín, 2013), and with van Dijk’s (1993a) description of racism as consisting of “properties attributed to the out-group . . . assumed to be inherently related to the racial or ethnic identity of the group” (p. 23). It corresponds with gendered expressions of cultural racism (Blaut, 1992; Bredström, 2003) directed toward immigrants and minority populations in Nordic countries including Sweden (Hervik, 2019; Keskinen, 2012; Mulinari & Neergaard, 2015; Saresma, 2019).

It may be added that not all of the articles mentioning “honor” included identifying information, and these therefore were not categorized according to perpetrator background. However, when the majority of articles using this term present the perpetrator as Other and in no case as Swedish, readers might still read Otherness into the other articles. This corresponds with de los Reyes’ (2003) argument that the word “immigrant” does not need to be mentioned for that to be what people hear in talk about “patriarchal values.”

Alongside the Nordic Paradox of coexisting high rates of IPVAW and of estimated gender equality (Gracia & Merlo, 2016), Hervik (2019) points to another paradox in Nordic countries, namely the coexistence of, on one hand, a self-image as tolerant, humane, and located outside of histories of colonialism and racism and, on the other hand, identification with the same basic division between the (civilized) self and the (uncivilized) Other that is found in colonial regimes (Keskinen et al., 2016). Correspondingly, an increasing intolerance toward immigrated groups coexists with claims of nonracism and references to cultural incompatibility (Hervik, 2019). Such evocation of cultural irreconcilability has taken expression not least through media representation of violence against women among immigrant groups (Hervik, 2019; Keskinen, 2012, 2016; Reimers, 2007), reminiscent of familiar, imperialist constructions of White persons rescuing White and non-White women from aggressive and sexually predatory men of foreign origin (Bredström, 2003; Spivak, 1988).

Although the noted differences in representation of IPVAW perpetrated by Swedish and non-Swedish-presenting men underscore the importance of directing further attention to how media representation shapes racialized thinking (e.g., Hervik, 2019) and affects population groups with immigrant backgrounds (Bredström, 2003), it can have implications for the targeting and implementation of IPVAW policies and interventions. Discourses construing IPVAW as located in Other cultures can not only further marginalize groups of people but are conducive to overlooking violence committed by White Europeans (Montoya & Rolandsen Agustín, 2013), while suggesting that nothing, in general, needs to change in Us—only in Them. Thus, possible connections and continuities between the IPVAW perpetrated by persons with minority status and the violence engaged in by nonimmigrants are concealed (Reimers, 2007). Meanwhile, othering of IPVAW may impede proper support measures from taking victims’ individual situations into consideration rather than relying on potentially discriminatory prejudices following ethnically and culturally stratified power relations (de los Reyes, 2003). A Swedish study (Eliassi, 2015) has shown that social workers culturalize and essentialize IPVAW to the extent that it is critical to their assessments of a situation, while upholding an assumption of universalism and color blindness. The media discourse examined in this study displays tendencies toward supporting such ideations. Meanwhile, construction of IPVAW as a non-Swedish issue can furthermore be conducive to victim-blaming, as women in Sweden are posited as not supposed to “allow” violence against them (Brännvall, 2016; de los Reyes, 2003).

At the same time, all IPVAW cases are not experienced in the same way (de los Reyes, 2003; Montoya & Rolandsen Agustín, 2013). Victims’ situations can depend on their position in society, and the experiences of members of different groups should be heard and taken seriously (e.g., de los Reyes, 2003; Montoya & Rolandsen Agustín, 2013). Keskinen (2011) warns not only against culturalization but also against universalizing assumptions that IPVAW dynamics are the same everywhere and against bypassing the IPVAW perpetrated or suffered by persons with immigrant backgrounds. An inclusionary, intersectional (Collins & Bilge, 2016; Crenshaw, 1989) approach is called for in the analysis of IPVAW discourses and interventions (Council of Europe, 2019; Montoya & Rolandsen Agustín, 2013; Wemrell et al., 2019). In addition, and crucially, culture should not be treated, in media representation or elsewhere, as essential or fixed conglomerates of static values or practices, but rather as contingent and complex, as encompassing various and potentially conflicting interpretations and expressions (Clifford, 1997; Gilroy, 1993), and as transversed by and involved in power relations (Brah, 1994; Bredström, 2003).

### Limitations

For the quantitative part of this study, a year’s worth of articles was gathered from newspapers from different parts of the country, yielding a broad data sample. The qualitative part encompassed a smaller time frame, yet still included a substantial number of articles. The selection and coding processes were meticulous.

Nevertheless, the study has its limitations. The search engine, Retriever Research, is somewhat unreliable in its presentation of search results as it sometimes skips a date, from which articles could be missed. This risk was minimized as our search was executed 3 times, and results from each search were compared with one another.

The categorization of types of discourses (structural, semi-structural, or individual) was not always clear-cut. Other researchers might have made different evaluations of some articles, which could potentially have a minor effect on the results. It should also be noted that information about the perpetrator’s national/ethnic/religious/cultural background was unavailable in most articles. The noted difference between discursive representations of IPVAW depending on the background of the perpetrator is thus based on limited material. Furthermore, the categorization of the alleged perpetrators as Swedish- or non-Swedish-presenting based on names or images was subjective, although done separately by two coauthors, and rested on our assumptions of what names and images were likely to be interpreted by readers as Swedish- or non-Swedish-presenting.

The nature of explanatory models found in this study should further be regarded in terms of an indication rather than a certainty, as it is possible that a larger sample would yield a somewhat different content and prevalence of categorizations.

As different newspapers sometimes report on the same cases of IPVAW, we knew on the basis of information provided elsewhere that some articles referred to IPVAW, even though they failed to establish a relationship between the victim and the perpetrator and therefore did not meet the inclusion criteria. Such a failure to define violent events as IPVAW in news reporting is interesting in itself.

Finally, this study points toward overall discursive tendencies in Swedish newspaper articles and does not include comparison between newspapers of different types or political affiliations. Such differences noted in our preliminary analysis pertained mainly to debate articles, the number of which were quite few in our present sample. An analysis of such differences represents an interesting potential objective for future research.

## Conclusion

High prevalence rates of IPVAW in Sweden and neighboring Nordic countries (e.g., FRA, 2014; Nybergh et al., 2013) remain a pressing concern and are seemingly paradoxical (Gracia & Merlo, 2016) due to the high levels of estimated gender equality in these countries. Although this study does not resolve this Nordic Paradox (Gracia & Merlo, 2016), it shows that despite the assumed link between gender inequality and IPVAW (e.g., UN, 2018), an individualizing discourse on IPVAW was found to be more predominant in Swedish newspapers than more structural conceptions of IPVAW, such as those associated with feminist and women’s shelter movements (e.g., SOU, 2004:121). Meanwhile, tendencies toward using a more structural discourse were stronger when the perpetrator was represented as non-Swedish. This confirms the importance of continued efforts toward public discourses that do not disregard the relevance of gendered relationships of power for IPVAW in Sweden or its high prevalence in the country. It also underscores the importance, not least for media reporting which likely affects public understanding and action, of avoiding stereotypes perpetuating ethnical/cultural/religious power relations in representations of IPVAW, as these may render IPVAW in the majority population invisible (de los Reyes, 2003), while aligning with xenophobic anti-immigration discourses (Eliassi, 2015). Although it is important to recognize the complexity of IPVAW causation, encompassing individual, relational, and structural factors (Heise, 1998), the structural dimensions of the commonly occurring societal phenomenon of IPVAW should not be overlooked.
